# Characterizing the Patterns of Electronic Health Record–Integrated Secure Messaging Use: Cross-Sectional Study

**DOI:** 10.2196/48583

**Published:** 2023-10-06

**Authors:** Laura R Baratta, Derek Harford, Christine A Sinsky, Thomas Kannampallil, Sunny S Lou

**Affiliations:** 1 Division of Biology and Biomedical Sciences Washington University School of Medicine Saint Louis, MO United States; 2 Department of Anesthesiology Washington University School of Medicine Saint Louis, MO United States; 3 American Medical Association Chicago, IL United States

**Keywords:** clinical care, clinician burden, communication, electronic health record, EHR, interprofessional communication, medical assistant, messaging, nurses, observational study, physicians, secure messaging, users

## Abstract

**Background:**

Communication among health care professionals is essential for the delivery of safe clinical care. Secure messaging has rapidly emerged as a new mode of asynchronous communication. Despite its popularity, relatively little is known about how secure messaging is used and how such use contributes to communication burden.

**Objective:**

This study aims to characterize the use of an electronic health record–integrated secure messaging platform across 14 hospitals and 263 outpatient clinics within a large health care system.

**Methods:**

We collected metadata on the use of the Epic Systems Secure Chat platform for 6 months (July 2022 to January 2023). Information was retrieved on message volume, response times, message characteristics, messages sent and received by users, user roles, and work settings (inpatient vs outpatient).

**Results:**

A total of 32,881 users sent 9,639,149 messages during the study. Median daily message volume was 53,951 during the first 2 weeks of the study and 69,526 during the last 2 weeks, resulting in an overall increase of 29% (*P*=.03). Nurses were the most frequent users of secure messaging (3,884,270/9,639,149, 40% messages), followed by physicians (2,387,634/9,639,149, 25% messages), and medical assistants (1,135,577/9,639,149, 12% messages). Daily message frequency varied across users; inpatient advanced practice providers and social workers interacted with the highest number of messages per day (median 19). Conversations were predominantly between 2 users (1,258,036/1,547,879, 81% conversations), with a median of 2 conversational turns and a median response time of 2.4 minutes. The largest proportion of inpatient messages was from nurses to physicians (972,243/4,749,186, 20% messages) and physicians to nurses (606,576/4,749,186, 13% messages), while the largest proportion of outpatient messages was from physicians to nurses (344,048/2,192,488, 16% messages) and medical assistants to other medical assistants (236,694/2,192,488, 11% messages).

**Conclusions:**

Secure messaging was widely used by a diverse range of health care professionals, with ongoing growth throughout the study and many users interacting with more than 20 messages per day. The short message response times and high messaging volume observed highlight the interruptive nature of secure messaging, raising questions about its potentially harmful effects on clinician workflow, cognition, and errors.

## Introduction

Communication among health care professionals is instrumental in the delivery of safe, timely, and high-quality patient care [1-3]. Estimates from time-and-motion studies have shown that clinicians spend up to 50% of their time communicating with others to coordinate patient care [4-10]. Although face-to-face communication is preferred [11-13], it is often not possible due to geographical and spatial separations and other constraints associated with clinical work. As such, alternative modes of communication (eg, telephone, pager, and SMS text messaging) are frequently used for clinical communication, each with its advantages and disadvantages.

With the rapid adoption of mobile phones, personal communication patterns have seen a dramatic shift toward the use of asynchronous, text-based communication [14]. There has been a corresponding shift in clinical communication toward secure messaging apps, with a recent survey-based study suggesting a doubling of secure messaging usage over the past 5 years [15]. The broad demand for secure messaging is further highlighted by the variety of available messaging tools, ranging from stand-alone mobile apps (eg, Voalte and TigerConnect) to electronic health record (EHR)–integrated solutions (Epic Secure Chat and Cerner CareAware) [3,15-17]. Despite the rapid adoption of secure messaging, relatively little is known about its use in clinical communication. Previous studies have largely focused on specific clinical units (eg, emergency rooms [18]) and have primarily characterized clinician behaviors and attitudes toward their usage [3,15,16,19-24].

We conducted a cross-sectional study on the usage patterns for Epic’s messaging system, Secure Chat, over a 6-month period within a large US health care system to characterize messaging frequency, usage across various types of health care professionals, message response latency, and messaging partners. We discuss the implications of secure messaging on clinician workflow, including its effects on work fragmentation, cognitive load, and potential downstream patient safety outcomes.

## Methods

### Study Setting and Design

This cross-sectional study was conducted at 14 hospitals and 263 outpatient clinics affiliated with Washington University and BJH Healthcare. These hospitals and clinics encompass both academic and community practice settings and serve a racially and socioeconomically diverse rural, suburban, and urban population across Missouri and Illinois. Epic (Epic Systems) was the EHR system that was used across all sites.

Epic’s Secure Chat messaging platform was launched across all clinical sites in September 2019. Secure Chat is a secure messaging platform that is embedded within the EHR, allowing users (ie, clinicians and support staff) to send messages to other users within the institution. Both mobile and desktop versions are available. On the desktop version, new messages appear as popups in the lower right-hand corner of the screen. On the mobile app, new messages are indicated through push notifications; to read a message, users must open Epic’s mobile app. Messages in both versions are threaded as conversations and a patient identifier can be optionally attached to a conversation to facilitate direct access to the patient chart.

### Data Collection

Data were collected on Epic’s Secure Chat usage from July 12, 2022, to January 17, 2023, from Epic’s Clarity database. For each message, information was collected on the time the message was sent, the conversation thread to which the message belonged, the sender of the message, the recipients of the message, the time at which the message was responded (if responded), and the message length in characters. Message content was not retrieved or used for this study. Additional metadata on users (ie, senders and recipients of messages) were also extracted from the EHR database, including the user’s role (ie, clinician type) and most recent practice location (ie, inpatient unit or outpatient clinic).

### Data Processing

#### Health Care Professional Categorization

A total of 74 unique user roles were categorized into user types by a clinician member of the study team into the following: pharmacist, physician, advanced practice provider (APP; ie, nurse practitioner or physician assistant), therapist (physical, occupational, or speech-language), medical assistant or technician, nurse, social worker or case manager, or other (mostly users with undefined user types in the system; Table S1 in Multimedia Appendix 1 for a full list of user types included in this category). Practice locations were categorized as either inpatient or outpatient, with emergency medicine categorized as an inpatient setting.

#### Message-Level Metrics

Message-level metrics were aggregated at the user level and the day level. Days were defined as 24-hour periods (midnight to midnight) during which the user sent or received at least one message; due to a lack of access to scheduling information, we were unable to account for workdays during which no messaging occurred. These user-day-level measures were aggregated across each user type, stratified by practice setting (inpatient vs outpatient), to create the following metrics for each user role and setting: number of unique secure messaging users, total messages sent, message length in characters, number of messages sent per day, and messages received per day.

#### Conversation-Level Metrics

As individual messages were threaded into conversations, the following conversation-level metrics were computed: number of users per conversation, number of messages per conversation, number of turns per conversation (where a turn is defined as a user sending one or more messages before a response by a second user), conversation duration (defined as the difference in time between the first and last messages in each conversation), and response latency (defined as the time difference between the first message from a user and a response by a second user).

Because most conversations (>80%) were between 2 users (ie, dyadic), additional analysis of messaging partners was conducted for dyadic conversations. For each user, the proportion of messages sent to their primary (ie, most frequent) messaging partner was also computed.

### Statistical Analysis

Descriptive statistics were calculated for categorical variables as count and percentage and for continuous variables as median and IQR. The Mann-Whitney *U* test was used to compare differences in continuous message-level metrics between inpatient and outpatient settings, whereas the chi-square test was used for categorical metrics. Bonferroni-corrected 2-tailed *P* values were reported following correction for multiple hypothesis testing; *P* values less than .05 were considered to be statistically significant. Data processing was performed using Python 3.9.7 (Python Software Foundation), and statistical analysis was conducted using R 4.2.2 (R Foundation for Statistical Computing) [25,26].

### Ethics Approval

This study was approved by the Washington University Institutional Review Board with a waiver for informed consent (IRB #202205084).

## Results

### Overall Messaging Patterns

A total of 32,881 users sent 9,639,149 messages across 1,547,879 conversations during the study period (Table 1; Table S2 in Multimedia Appendix 1). Message volume steadily increased throughout the study (Figure S1 in Multimedia Appendix 1); median message volume was 53,951 (IQR 30,871-55,537) messages per day during the first 2 weeks of the study period and 69,526 (IQR 32,023-73,237) messages per day during the last 2 weeks, resulting in an overall increase of 29% (*P*=.03).

**Figure 1:**
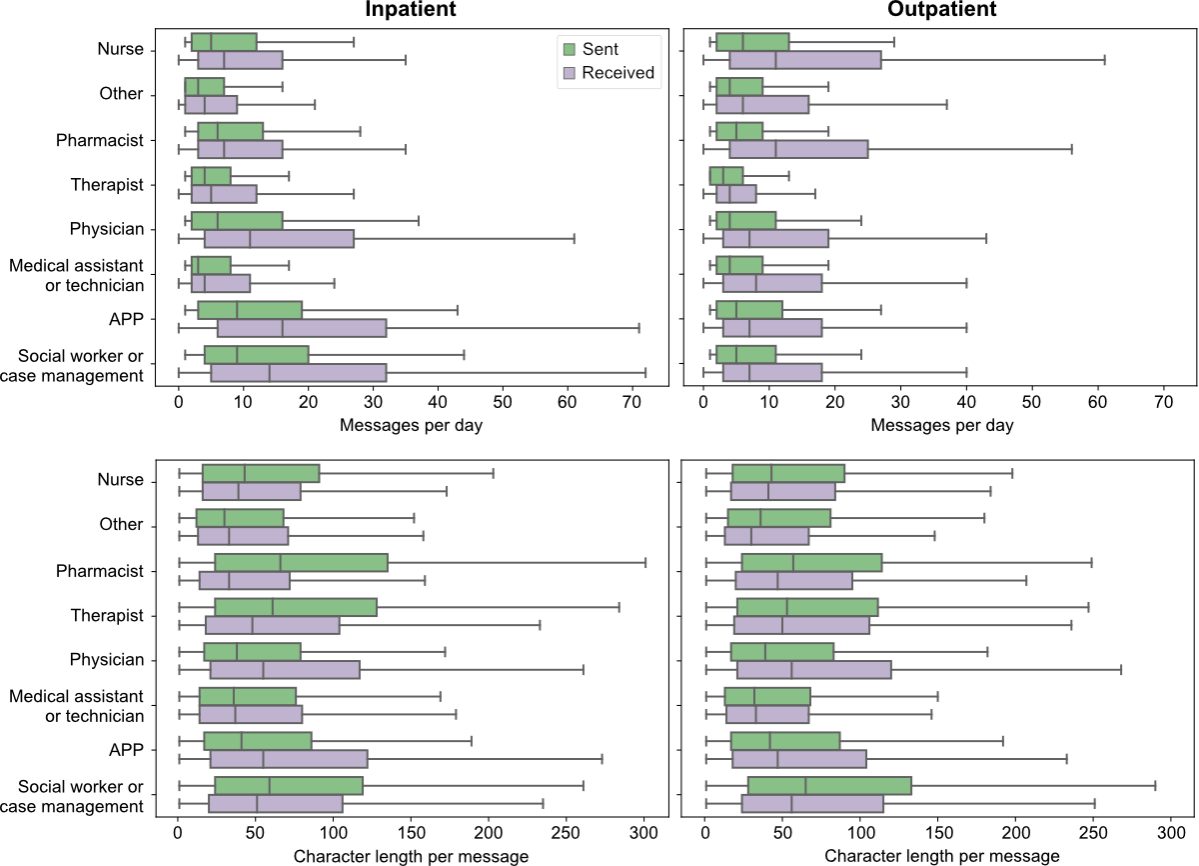
Box plots illustrating the distribution of secure messaging behaviors stratified by user role and inpatient versus outpatient setting. The top panel shows the daily volume of messages sent (green) and received (purple) by each clinician group in the inpatient (left) and outpatient (right) settings. The bottom panel shows the median message length for sent (green) and received (purple) messages. APP: advanced practice provider.

Messaging patterns differed by clinician role and practice setting (*P*<.001; Figure 1). In inpatient settings, nurses were the most frequent users (3,122,769/6,371,703, 49% messages), followed by physicians (1,380,276/6,371,703, 22% messages), and APPs (565,134/6,371,703, 9% messages). In outpatient settings, physicians sent 1,007,358 (31%) of 3,252,968 messages, followed by medical assistants (859,730/3,252,968, 26%) and nurses (761,501/3,252,968, 23%).

Inpatient APPs and social workers had the highest median daily number of messages (top part of Figure 1); they sent a median of 9 (IQR 3-20) and 9 (IQR 4-20) messages per day and received a median of 10 (IQR 3-23) and 10 (IQR 3-27) messages per day, respectively. The median number of messages received by all users was 6 (IQR 3-16) and 6 (IQR 2-16) for inpatient and outpatient settings, respectively.

**Table 1:** Secure messaging usage patterns stratified by health care professional type and inpatient versus outpatient setting.

User category	Inpatient							Outpatient										
	Users using secure chat, n (%)	Total messages sent, n (%)	Messages sent per day, median (IQR)	Character length per message, median (IQR)	Messages received per day, median (IQR)	Character length, received, median (IQR)	Users using secure chat, n (%)		Messages sent, n (%)		Messages sent per day, median (IQR)		Character length per message, median (IQR)		Messages received per day, median (IQR)		Character length received, median (IQR)	
APP^a^	926 (5)	565,134 (9)	9 (3-19)	41 (17-86)	10 (3-23)	55 (21-122)	755 (7)		347,008 (11)		5 (2-12)		42 (17-87)		6 (2-15)		47 (18-104)	
Medical assistant or technician	2429 (12)	275,847 (4)	3 (2-8)	36 (14-76)	4 (1-10)	37 (14-80)	3462 (33)		859,730 (26)		4 (2-9)		32 (13-68)		6 (2-16)		33 (14-67)	
Nurse	10,289 (50)	3,122,769 (49)	5 (2-12)	43 (16-91)	6 (2-15)	39 (16-79)	1863 (18)		761,501 (23)		6 (2-13)		43 (18-90)		8 (3-22)		41 (17-84)	
Pharmacist	452 (2)	253,854 (4)	6 (3-13)	66 (24-135)	6 (2-14)	33 (14-72)	110 (1)		33,886 (1)		5 (2-9)		57 (24-114)		8 (3-21)		47 (20-95)	
Physician	2988 (15)	1,380,276 (22)	6 (2-16)	38 (17-79)	8 (2-21)	55 (21-118)	2712 (26)		1,007,358 (31)		4 (2-11)		39 (17-83)		5 (2-15)		56 (21-120)	
Social worker	354 (2)	313,444 (5)	9 (4-20)	59 (24-119)	10 (3-27)	51 (20-106)	100 (1)		19,052 (0.6)		5 (2-11)		65 (28-133)		6 (2-15)		56 (24-115)	
Therapist	1401 (7)	276,901 (4)	4 (2-8)	61 (24-128)	4 (2-10)	48 (18-104)	441 (4)		39,181 (1)		3 (1-6)		53 (21-112)		3 (1-7)		50 (19-106)	
Other	1577 (8)	183,478 (3)	3 (1-7)	30 (12-68)	3 (1-8)	33 (13-71)	1109 (11)		185,252 (6)		4 (2-9)		36 (15-81)		5 (2-14)		30 (13-67)	
Total	20,503 (100)	6,371,703 (100)	5 (2-12)	43 (17-91)	6 (2-16)	42 (17-89)	10,582 (100)		3,252,968 (100)		4 (2-11)		39 (16-82)		6 (2-16)		41 (16-88)	

^a^APP: advanced practice provider.

The median character length of sent messages was 41 (IQR 17-88) for all messages sent across all users and settings (bottom part of Figure 1). Inpatient pharmacists (median length 66, IQR 24-135 characters) and outpatient social workers (median length 65, IQR 28-133 characters) sent the longest messages. Most user types, on average, received messages that were similar in length to those that they sent (Table 1). However, physicians received messages that were longer than those that they sent across inpatient and outpatient settings, while inpatient pharmacists and therapists typically sent longer messages than they received (*P*<.001 for all comparisons).

A total of 6,649,198 (69%) of 9,639,149 messages were associated with a specific patient’s chart. It was more common for messages sent from the inpatient setting (4,7815,457/6,371,703, 74% messages) to include an attached patient chart compared to the outpatient setting (1,933,265/3,252,968, 59% messages; *P*<.001).

### Conversation Dynamics

Most conversations were between 2 users (1,258,036/1,547,879, 81% conversations). Overall, 181,971 (12%) of 1,547,879 conversations were between 3 users, and 107,872 (7%) of 1,547,879 conversations were between 4 or more users. Conversations were generally short, with a median of 4 (IQR 2-7) messages per conversation and 2 (IQR 1-4) conversational turns (Table 2).

Conversations lasted a median of 25 (IQR 4.9-235) minutes. For conversations with responses, the median response time was 2.4 (IQR 0.65-15) minutes. Inpatient pharmacists had the quickest median response times (1.4, IQR 0.5-5.6 minutes), followed by inpatient APPs at 1.9 (IQR 0.6-10.3) minutes, outpatient pharmacists at 2.1 (IQR 0.6-9.0) minutes, and outpatient nurses at 2.3 (IQR 0.7-14.5) minutes (Table S3 in Multimedia Appendix 1).

**Figure 2:**
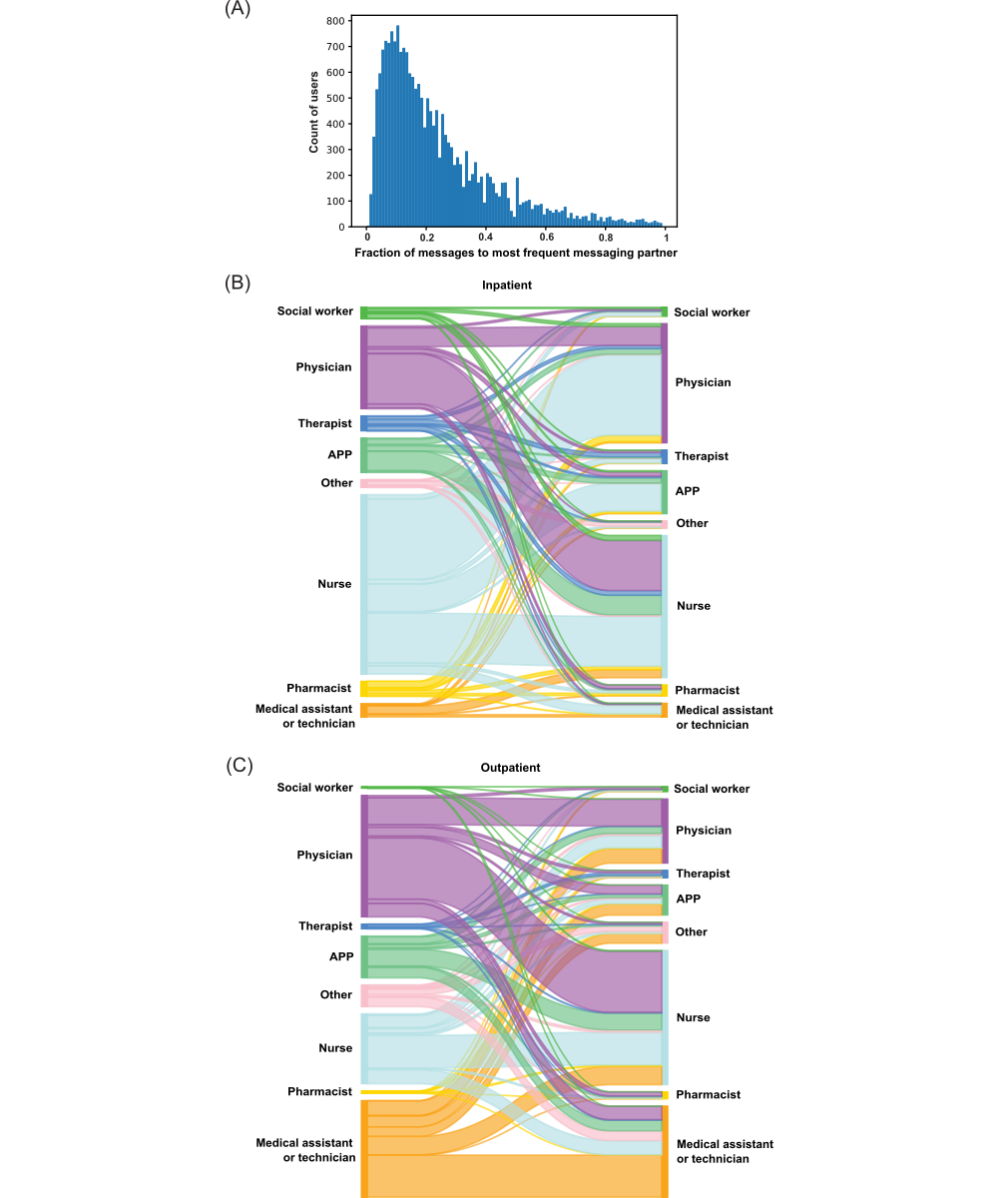
Characteristics of secure messaging partners. (A) Histogram showing, for each user, the fraction of messages sent to each user’s most frequent messaging partner. Sankey plots illustrate the volume of messages between each user type with a sender (left) and receiver (right) pair in (B) inpatient versus (C) outpatient settings. APP: advanced practice provider.

We examined dyadic conversations in greater detail. The median number of dyadic messaging partners for each user was 14 (IQR 4-43). Inpatient users had a greater number of dyadic messaging partners (median 18, IQR 4-52) than outpatient users (median 10, IQR 3-26; *P*<.001). Users sent a median of 18% (IQR 10%-33%) of their messages to their most frequent dyadic messaging partner (Figure 2A).

The largest proportion of inpatient dyadic messaging volume was from nurses to physicians (972,243/4,749,186, 20% messages), followed by physicians to nurses (606,576/4,749,186, 13% messages; Figure 2B). The largest proportion of outpatient dyadic messaging volume was from physicians to nurses (344,048/2,192,488, 16% messages), followed by medical assistants sending messages to other medical assistants (236,694/2,192,488, 11% messages; Figure 2C).

**Table 2:** Characteristics of secure messaging conversations.

Characteristics		Value
**Users per conversation, n (%)**		
	2	1,258,036 (81.3)
	3	181,971 (11.8)
	>4	107,872 (6.98)
Messages per conversation, median (IQR)		4 (2-7)
Conversation duration (minutes), median (IQR)		25 (4.9-235)
Number of turns per conversation, median (IQR)		2 (1-4)
Response latency (minutes), median (IQR)		2.4 (0.65-15)

## Discussion

### Principal Findings

In this cross-sectional study of EHR-integrated secure messaging among >30,000 users across 14 hospitals and >250 outpatient clinics, we found that weekly message volume increased approximately 31% over a 6-month period and that secure messaging was widely used in both inpatient and outpatient settings by a diverse range of health care professionals. The daily volume of secure messaging was high, with many users sending or receiving upwards of 20 messages per day.

The main strength of this study is its scope, namely that we measured secure messaging use across a diversity of hospital and clinic settings and health care professional groups. Although others have characterized secure messaging use in individual inpatient units or clinics [23,27-31], this study is one of the first to describe secure messaging use across an entire health care organization. We found that secure messaging is widely used by many different health care professionals in both inpatient and outpatient contexts. In addition, we found that for many users, the daily volume of secure messages was comparable to the number of telephone calls or pager messages received by physicians reported in other studies [32,33]. These results illustrate the essential role that secure messaging plays in interprofessional communication across the organization.

### Limitations

However, this study also has several limitations. First, because we were unable to access work schedules, measurement of secure messaging behavior was limited to days in which the user sent or received a message; workdays in which the user did not use secure messaging were not included in the analysis. In addition, users who did not use secure messaging during the study period were not included. Thus, these results should be interpreted as only representative of secure messaging behaviors for user days in which secure messaging was in active use. Second, this study was conducted at a single health care system; although many clinic sites and hospitals were included, these results may be partially influenced by local culture, variable adoption, and the specific secure messaging platform, which may not generalize to other sites. Finally, because the content of secure messages was not available, we can only speculate as to the motivations behind secure messaging use.

### Implications and Future Directions

Nonetheless, these results highlight the rapid growth and high volume of secure messaging as a form of clinical communication. Given that secure messaging continues to be increasingly adopted across the United States [15], further studies are needed on the impact of secure messaging on clinician workflow and its downstream effects on patient care. For example, secure messaging might improve connectivity by lowering the barrier to communication, potentially increasing care collaboration and efficiency. Because secure messaging is an asynchronous form of communication, its use might also reduce interruptions, with potential downstream benefits for patient safety [34-38].

However, it is unknown whether secure messaging replaces other modes of communication versus simply increasing the overall burden of communication [39]. For example, although it is intended to be used asynchronously, there may be a large amount of near-synchronous use (as evidenced by the median response time of ~2 minutes that we found), potentially resulting in increased interruptions and time spent managing communication [40-42]. In addition, secure messaging may be less efficient than synchronous face-to-face or telephone communication; we found that secure messaging conversations involved a median of 2 conversational turns spanning 25 minutes, and it is possible that many conversations could have been resolved with a shorter telephone call. Over a quarter of secure messages in this study did not have an associated patient chart, potentially increasing the workflow burden to address such messages (eg, additional work to identify the patient in question and navigate to their chart) and further contributing to workflow inefficiency.

Given the potential advantages and concerns surrounding secure messaging use described above, further research is needed on the impact of secure messaging on clinician efficiency, workflow, cognitive burden, and downstream effects for patient care and clinician wellness, as emphasized by the American Medical Informatics Association’s 25×5 campaign to reduce EHR burden [43]. In addition, no standardized guidelines exist for secure messaging use or deployment [16], and additional research is needed to understand the messaging policies (such as establishing etiquette and guidelines for appropriate use and best practices for education on these topics) and platform features (such as batched alerts, priority indicators, improved notification design, or generative artificial intelligence) that best enhance clinical care and minimize the potential negative effects of secure messaging use [44].

### Conclusions

We characterized the usage patterns of EHR-integrated secure messaging across a large health care consortium and found that it was used by a diverse range of health care professionals across inpatient and outpatient settings. In addition, the burden of secure messaging communication was relatively high, with the interruptive nature of secure messaging raising concerns regarding cognitive overwhelm and patient safety, highlighting the need for further research on the effects of secure messaging on clinician workflow and cognitive burden and the best messaging strategies that minimize that burden.

## Appendix

Multimedia Appendix 1 Supplemental figures and tables.

## Abbreviations

APP: advanced practice provider
EHR: electronic health record

## Data Availability

The data are not publicly available due to information that could compromise the privacy of study participants.
