# Supplementary material for: Characterizing the Patterns of Electronic Health Record–Integrated Secure Messaging Use: Cross-Sectional Study
Source: J Med Internet Res. 2023 Oct 6;25:e48583. doi: 10.2196/48583 (PMC10589827; doi:10.2196/48583)
Supplement: Multimedia Appendix 1 [file jmir_v25i1e48583_app1.docx]

**Figure S1**. Trends in the total volume of secure messages sent during the study period.


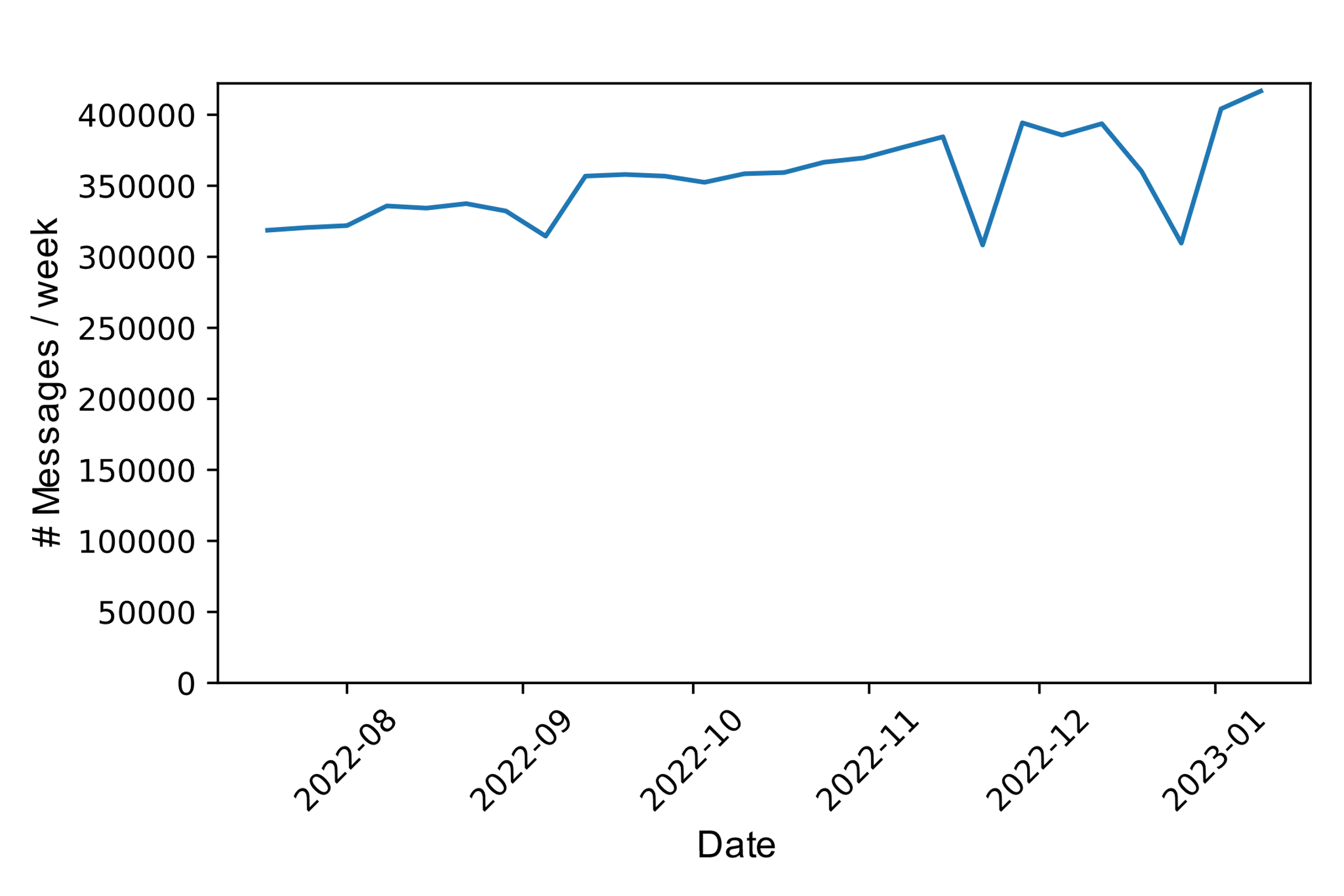


**Figure S2.** Volume of secure messages varies by day of week.


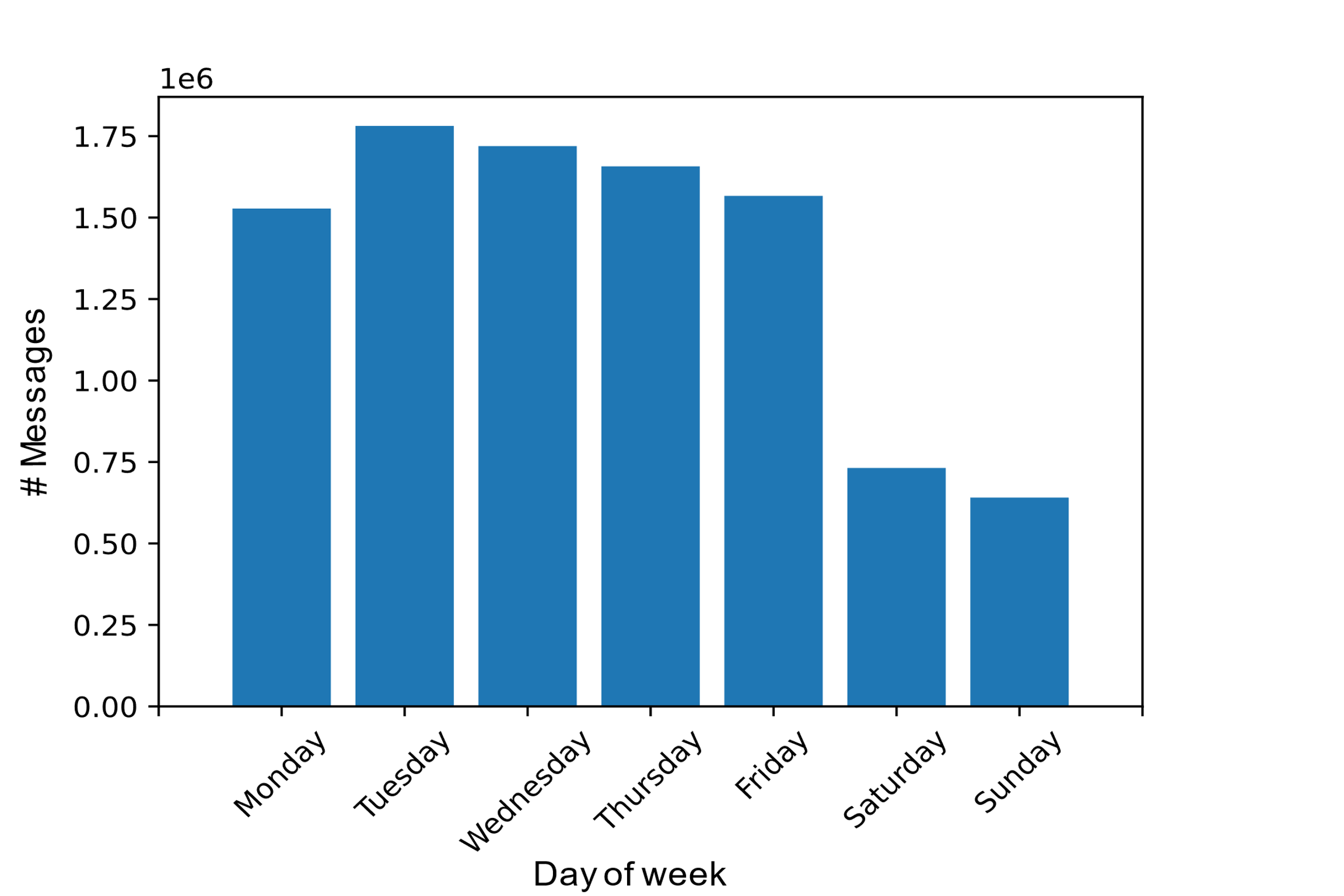


**Figure S3.** Distribution of conversation duration (left) and number of dyadic messaging partners (right), stratified by user type and inpatient (blue) versus outpatient setting (orange).


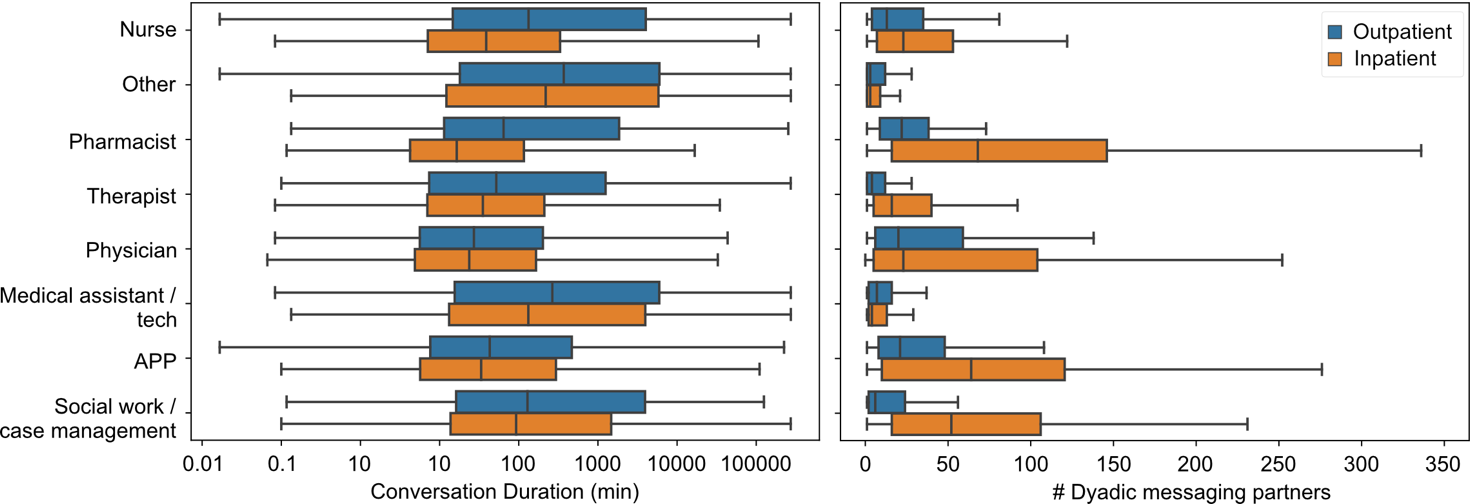


**Table S1.** Clinician roles grouped into the “Other” category with their respective user counts. The most common user grouped into this “Other” category were users with an undefined clinician role in the system; this could be related to the user having a nonclinical role, or the user being a temporary worker.

| **Provider Role** | **Count** |
| --- | --- |
| Undefined | 314729 |
| Interface User | 18806 |
| Research Team Member | 11533 |
| Paramedic | 9957 |
| Optometrist | 9835 |
| Audiologist | 5098 |
| Spiritual Care | 2155 |
| Community Health Navigator | 1977 |
| Psychologist | 1945 |
| Infection Preventionist | 1444 |
| Financial Advisor | 1417 |
| Counselor | 1299 |
| Health Educator | 912 |
| Utilization Manager | 668 |
| Midwife | 628 |
| Principal Investigator | 279 |
| Dentist | 111 |
| School Teacher | 82 |
| Perfusionist | 14 |
| Interpreter | 8 |
| Dental Assistant | 7 |
| Clinical Neurophysiologist | 7 |

**Table S2.** Secure messaging characteristics for all users in the study regardless of work setting, stratified by provider type.

| **Participants** | N (%) | Total messages sent; N (%) | Messages sent per day; median (IQ) | Character length per day; median (IQR) | Messages received per day; median (IQR) | Character length received; median (IQR) |
| --- | --- | --- | --- | --- | --- | --- |
| APP | 1,549 (5%) | 912,142 (9%) | 6 (2-16) | 41 (17-86) | 8 (2-20) | 53 (20-117) |
| Med assistant/tech | 5,689 (19%) | 1,135,646 (12%) | 4 (2-9) | 33 (13-70) | 6 (2-14) | 34 (14-70) |
| Nurse | 11,971 (41%) | 3,884,439 (40%) | 5 (2-12) | 43 (17-91) | 6 (2-16) | 39 (16-79) |
| Other | 2,702 (9%) | 382,911 (4%) | 3 (1-8) | 32 (13-72) | 3 (1-10) | 32 (13-69) |
| Pharmacist | 540 (2%) | 287,747 (3%) | 6 (3-12) | 64 (24-133) | 6 (2-15) | 36 (16-77) |
| Physician | 4,810 (16%) | 2,387,634 (25%) | 5 (2-13) | 38 (17-81) | 6 (2-18) | 55 (21-118) |
| Social worker | 437 (1%) | 332,496 (3%) | 8 (3-19) | 59 (24-120) | 9 (3-25) | 51 (21-107) |
| Therapist | 1,768 (6%) | 316,134 (3%) | 3 (2-7) | 60 (24-126) | 4 (2-9) | 49 (18-104) |
| **Total** | 29,466 | 9,639,149 | 5 (2-11) | 41 (17-88) | 6 (2-16) | 42 (17-88) |
| **Clinical setting** | N (%) |  |  |  |  |  |
| Inpatient | 14 |  |  |  |  |  |
| Outpatient | 268 |  |  |  |  |  |

**Table S3.** Response time to secure chat messages stratified by recipient provider type and setting. Mann-Whitney *U* tests were performed to compare the inpatient and outpatient response times for each provider type; p-values were adjusted by Bonferroni correction.

|  | **Inpatient** | **Outpatient** |  |
| --- | --- | --- | --- |
| **Provider category** | **Response time** (min) | **Response time** (min) | **p-value** |
| APP | 1.9 (0.6 - 10.3) | 2.4 (0.7-16.4) | <0.001 |
| Med assist/tech | 2.9 (0.7-31.8) | 2.7 (0.6-28.0) | <0.001 |
| Nurse | 2.5 (0.7-14.5) | 2.3 (0.7-14.5) | 0.008 |
| Other | 2.8 (0.6-36.8) | 2.7 (0.6-31.6) | 0.121 |
| Pharmacist | 1.4 (0.5-5.6) | 2.1 (0.6-9.0) | <0.001 |
| Physician | 2.1 (0.6-10.5) | 2.8 (0.7-16.7) | <0.001 |
| Social worker | 2.8 (0.8-18.6) | 2.9 (0.8-19.9) | 0.002 |
| Therapist | 2.7 (0.7-20.5) | 2.7 (0.7-29.6) | <0.001 |
